# Elevated Social Stress Levels and Depressive Symptoms in Primary Hyperhidrosis

**DOI:** 10.1371/journal.pone.0092412

**Published:** 2014-03-19

**Authors:** Katharina M. Gross, Andrea B. Schote, Katja Kerstin Schneider, André Schulz, Jobst Meyer

**Affiliations:** 1 Department of Neurobehavioral Genetics, Institute of Psychobiology, University of Trier, Trier, Germany; 2 Department of Psychology, Cognitive Psychology, University of Trier, Trier, Germany; 3 Sub-domain Self-Regulation and Health, Research Unit INSIDE, University of Luxembourg, Walferdange, Luxembourg; 4 Department of Clinical Psychophysiology, Institute of Psychobiology, University of Trier, Trier, Germany; University of Tennessee, United States of America

## Abstract

Primary hyperhidrosis is defined as excessive sweating of certain body areas without physiological reasons. Hyperhidrotic individuals report a high psychological strain and an impairment of their quality of life. Thus, the aim of the study is to investigate the relation between hyperhidrosis and different psychological as well as physiological aspects of chronic stress as a co-factor for the etiology of depression. In this study, forty hyperhidrotic subjects were compared to forty age- and sex-matched healthy control subjects. The Trier Inventory of Chronic Stress (‘*Trierer Inventar zum chronischen Stress’*: TICS), the Beck Depression Inventory (BDI-II) and the Screening for Somatoform Disorders (SOMS-2) were used to examine the correlation between primary hyperhidrosis and stress as well as accompanying depressive and somatic symptoms. The cortisol awakening response of each subject was analyzed as a physiological stress correlate. In hyperhidrotics, we found a significant lack of social recognition as well as significantly more depressive symptoms compared to the control subjects. A subgroup of patients with axillary hyperhidrosis had the highest impact on these increased issues of chronic stress, pointing to a higher embarrassment in these subjects. Especially in social situations, hyperhidrotics showed higher stress levels, whereby a vicious circle of stress and sweating is triggered. However, the cortisol awakening response did not significantly differ between hyperhidrotics and controls. Moreover, affected persons suffer from more depressive symptoms, which may be caused by feelings of shame and a lack of self-confidence. This initial study provides an impetus for further investigation to reveal a causative relationship between hyperhidrosis and its psychological concomitants.

## Introduction

Sweating, for example in Finnish Sauna or Turkish Hammam, is accepted as a form of promoting well-being and social interaction. However, in everyday life, it is associated with lacking hygiene and classified as a social *no-go*. Furthermore, it is often accompanied by a high psychological strain and, in worst cases, even social isolation [1]. In general, sweating can be divided into thermoregulatory and emotional sweating, both regulated by different physiological mechanisms [2]. People affected with excessive emotional and increased thermoregulatory sweating, which exceed physiologically necessary levels, are diagnosed as hyperhidrotic [3], [4].

Hyperhidrosis is separated into a primary and a secondary form. Primary hyperhidrosis, which was investigated in this study, is characterized by excessive sweating of particular body regions as a disease pattern *per se*. Although objective measurements of the absolute sweat secretion can be performed, the impact of hyperhidrosis and its diagnosis highly depend on the individual perception and hence the psychological strain of the affected person. In the ICD-10, localized hyperhidrosis is characterized as R61.0. The mainly affected regions are the palms, the plants, the armpits and the face. The prevalence in the U.S. is about 2.8% for primary hyperhidrosis, whereof one half of the patients suffer from axillary hyperhidrosis [5]. Palmar hyperhidrosis, which affects approximately 0.6 to 1% of the Western population, is often related to plantar hyperhidrosis [6]. Excessive sweating usually has its onset in adolescence. However, hyperhidrosis of palms and plants often already occurs in childhood [7].

The skin is the major organ in the sweating process and a barrier between the external and internal environment. It is made up of three different dermal layers: epidermis, dermis – with the adnexal structures including, amongst others, the sweat glands – and subcutaneous tissue [8]. Epidermal, dermal and adnexal structures communicate through the production of neurotransmitters, neuropeptides, neurohormones and hormones to regulate the local homeostasis. Likewise, skin cells express receptors for neuropeptides and neurotransmitters similar to those expressed in the central neuroendocrine systems. Therewith, the skin is a potential target of neural responses [9]. Moreover, the skin transfers sensory stimuli via afferent nerves to the central nervous system, where the thalamus as a central coordinator receives and sends neural signals to the central structures of the autonomic nervous system [10].

As a putative cause of primary hyperhidrosis, the highly increased sympathetic activity of the autonomic nervous system is postulated [11]. Eccrine glands are, via the neurotransmitter acetylcholine, innervated by postgaglionic sympathetic fibres and lead to a subsequent increase of sweat secretion. However, apocrine glands respond to emotional stimuli and are activated via innervation or via circulating catecholamines. Apoeccrine glands are mixed type glands and show a greater responsiveness to cholinergic and adrenergic stimuli than eccrine glands. They have a very high overall sweat rate and may contribute strongly to axillary sweating [12]. Since a sympathetic overactivity has also been shown in patients with somatization disorders [13], we were interested in the relationship of somatic symptoms and hyperhidrosis in affected persons.

In contrast to the primary form, secondary hyperhidrosis is characterized by excessive sweating caused by a variety of physiological conditions. These include, amongst others, obesity, menopause, infections, intoxication, malignant tumors and endocrinologic, cardiovascular or neurologic dysfunctions as well as certain medications. Most of the patients with secondary hyperhidrosis sweat generalized all over their body [7].

In this study, we focus on self-perceived chronic psychological stress in persons affected by hyperhidrosis, which has a strong social component. In general, the body of a human being adapts successfully to environmental and internal challenges, which lead to complex physiological, psychological and behavioral responses. This stress response depends on the situation, the kind of stressor and whether the stress is acute, episodic or chronic. Avoiding a handshake, wearing dark clothes, changing shirts several times a day, worrying about someone seeing the pit stains – all of this implies that psychological stress constitutes a strong social component in the everyday life of the hyperhidrotics [14]. Although there is evidence that affected persons suffer from a decreased quality of life only few studies have been performed [1], [15]. Under discussion are the lack of self-confidence and bodily discomfort as well as feelings like shame and fear as mediators of disturbed interpersonal relationships [5] and social seclusion [16]. These mental states cause even more psychosocial stress, which represents a considerable risk factor for the development of many diseases and disorders, such as depression. Finally, the development of depressive symptoms observed in some hyperhidrotics [17] might be triggered by a vicious circle of sweating and psychosocial stress.

Psychosocial stress – as one possible issue in hyperhidrosis – leads to the activation of the hypothalamic-pituitary-adrenal (HPA) axis. The secretion of corticotropin-releasing hormone (CRH) and arginine-vasopressin in the hypothalamus leads to the production of adrenocorticotropic hormone (ACTH) in the anterior pituitary, which results in the secretion of cortisol from the adrenal glands. Cortisol as the major stress hormone acts on hypothalamus, pituitary and hippocampus and inhibits its own production by a negative feedback loop. Interestingly, the skin has its own stress response system providing local protection for the skin and internal homeostasis [18]. This cutaneous HPA axis is activated by physiological stressors, such as bursts of radiation, mechanical energy or chemical and biological insults [9]. Probably, the skin can regulate the central HPA axis depending on the intensity and nature of the stressor [8]. Furthermore, the skin produces all peptide hormones being central constituents of the HPA axis [9]. CRH, for example, is not only synthesized in the hypothalamus in consequence of an activated HPA axis, but also in the skin itself. Moreover, CRH and its receptor CRHR1 were found in sweat glands [8]. The systemic HPA axis shows an increase in the cortisol response to awakening (cortisol awakening response, CAR) [19], which has been widely used as a sensitive and stable measurement for the physiological reactivity of the HPA axis under baseline conditions [19]. Thus, the CAR is not a measure for acute but rather for high chronic stress. In meta-analyses, an increased CAR has been linked to job and general life stress, whereas a decreased CAR was correlated with fatigue, burnout or exhaustion [20]. The effects of stress-related factors on the CAR may actually depend on the duration of stressful conditions. Chronic stress might initially be associated with an increased HPA axis activity, whereas individuals can develop a HPA axis hypoactivity if chronic stress persists for a longer period of time [21], [22]. CAR has been thoroughly investigated with regard to depression revealing opposing results [20]. However, so far, no study has ever addressed the correlation of CAR and hyperhidrosis.

We hypothesize that, due to the burden of their disorder, the hyperhidrotic subjects of our sample perceive more chronic psychosocial stress compared to the normohidrotic control group. Based on this, we assume that the hyperhidrotic group suffers from more depressive symptoms than the controls as a result of the permanent exposure to psychosocial stress. Furthermore, for our group of hyperhidrotic subjects, we hypothesize a decreased CAR as a measure of HPA reactivity caused by very high chronic stress levels. Finally, increased stress levels are accompanied by other symptoms induced by the autonomic nervous system. Thus, it can be assumed that hyperhidrotic persons show more somatic symptoms compared to the control subjects. This is the first study focusing on hyperhidrosis and psychosocial stress by examining particular facets of chronic stress.

## Materials and Methods

### Ethics Statement

This study was approved by the Ethics Committee of the University of Trier according to *The Code of Ethics of the World Medical Association (Declaration of Helsinki) for experiments involving humans*. All participants gave a written informed consent that they took part in the study voluntarily.

### Sampling Techniques and Participants

We contacted local dermatologists and general practitioners to recruit an age- and sex-matched sample consisting of 40 subjects excessively sweating on palms, plants and/or armpits and 40 healthy control subjects. The age of the hyperhidrotic subjects was between 19 and 72 years (*M*: 31.43; *SD*: 14.34) and the age of the control subjects was between 19 and 73 years (*M*: 31.83; *SD*: 14.58). The sample consisted of university students, employees and unemployed persons. All subjects were of European descent. The mean Body Mass Index (BMI) of the excessively sweating subjects was 24.49 kg/m^2^ (range: 17.63 to 41.12; *SD*: 5.37) and the one of the control group was 23.22 kg/m^2^ (range: 19.05 to 30.42; *SD*: 2.84). An overview is given in Table 1. Persons with mental, endocrinological or metabolic disorders potentially indicating secondary hyperhidrosis were excluded from our sample. Furthermore, subjects were not allowed to use any medication except oral contraceptives. Additionally, women taking the oral contraceptives *Yasmin, Yasminelle* or *Petibelle* were excluded because of the component drospirenone, an antagonist of the mineralcorticoid receptor, which may affect the stress reactivity of the body through a modified cortisol release [23].

**Table 1:** Specified description of the sample.

	Hyperhidrotic subjects	Control subjects	Statistic	*p*	Effect size φ
age [mean (*SD*)]	31.43 (14.34)	31.83 (14.58)	*t*(78) = 0.124	0.902	
age [N (%)]					
19 to 30	28 (35%)	28 (35%)	?^2^(1) = 0	1.000	0.000
31 to 50	6 (7.5%)	6 (7.5%)	?^2^(1) = 0	1.000	0.000
51 to 73	6 (7.5%)	6 (7.5%)	?^2^(1) = 0	1.000	0.000
sex [N (%)]					
-male	20 (25%)	20 (25%)	?^2^(1) = 0	1.000	0.000
-female	20 (25%)	20 (25%)	?^2^(1) = 0	1.000	0.000
BMI [mean (*SD*)]	24.49 (5.37)	23.22 (2.84)	*t*(78) = 1.325	0.189	
BMI [N (%)]					
<19	2 (2.5%)	0 (0%)	?^2^(1) = 0	1.000	0.000
19 to 24	22 (27.5%)	27 (33.75%)	?^2^(1) = 1.317	0.251	0.128
>24	16 (20%)	13 (16.25%)	?^2^(1) = 0.487	0.485	0.078
employment [N (%)]					
-student	29 (36.25%)	29 (36.25%)	?^2^(1) = 0	1.000	0.000
-employed	9 (11.25%)	8 (10%)	?^2^(1) = 0.075	0.785	0.031
-unemployed	2 (2.5%)	3 (3.75%)	?^2^(1) = 0.231	0.644	0.052

Notes: BMI: Body Mass Index; *SD*: Standard deviation.

### Procedures and Measures

All individuals were carefully screened by a trained psychologist (K.M.G.). They underwent a medical and psychological anamnestic screening to gather information about their present and past medical status. Those individuals complaining about excessive sweating, who did not yet consult a dermatologist (30 out of 40 hyperhidrotic subjects), underwent a gravimetric measuring procedure of their sweat production. For this purpose, weighted filter paper was pressed on the sweating body area and weighted after one minute [24]. All participants exceeded the threshold values of 30 mg/min for palms and 50 mg/min for armpits, respectively [25]. All hyperhidrotic subjects filled in parts A and B of the German version of the Hyperhidrosis Impact Questionnaire (HHIQ) [26], to provide information about the onset, the affected areas of the body and the extent of sweating of the respondents. Furthermore, the German versions of the following questionnaires were used: (1) The Trier Inventory for Chronic Stress (*Trierer Inventar zum chronischen Stress*: TICS) [27] consists of nine subscales, which measure the amount of self-reported chronic stress. (2) By means of the Beck Depression Inventory (BDI-II) [28] (German version: [29]), depressive symptoms can be revealed by counting up the single item values to a total sum. (3) The Screening for Somatoform Disorders (SOMS-2) [30] serves as a self-report measure for somatic symptoms. All questionnaires are reliable and validated measuring instruments for the German market [27], [29], [30] and are summarized in Table 2.

**Table 2:** Means and standard deviations (in parentheses) of hyperhidrotic, axillary hyperhidrotic and control subjects for different questionnaires and cortisol.

	Hyperhidrotic subjects N = 40	Axillary hyperhidrotic subjects N = 20	Control subjects N = 40	Difference (Hyperhidrosis - Control subjects) F(1,78)/p	Difference (Axillary hyperhidrosis -Control subjects) F(1,58)/p
TICS					
-work overload	55.18 (11.50)	59.00 (12.69)	50.78(12.14)	2.77/0.100	5.75/0.020*
-social overload	52.55 (8.59)	53.95 (7.66)	48.30 (7.22)	5.74/0.019*	7.58/0.008*
-pressure to succeed	49.75 (8.62)	51.32 (9.09)	47.58 (7.86)	1,39/0.242	2.64/0.110
-work discontent	56.10 (8.75)	58.79 (7.61)	52.72 (8.11)	3.20/0.078	7.49/0.008*
-excessive demands from work	59.60 (12.07)	64.58 (9.81)	54.33 (9.24)	4.82/0.031*	15.27/0.00025**
-lack of social recognition	56.07 (11.52)	60.11 (9.19)	48.13 (9.04)	11.79/0.001**	22.38/0.000015**
-social tensions	51.38 (11.26)	53.21 (13.56)	48.70 (10.96)	1.16/0.285	1.87/0.177
-social isolation	53.73 (9.93)	56.53 (10.60)	51.75 (10.81)	0.73/0.397	2.55/0.116
-chronic worrying	56.03 (10.93)	60.89 (9.76)	50.98 (8.30)	5.42/0.023*	16.42/0.000156**
-chronic stress screening scale	58.23 (11.87)	63.26 (10.26)	52.28 (7.89)	6.98/0.010*	20.50/0.000031**
BDI-II	10.53 (8.71)	14.26 (8.93)	4.83 (3.13)	15.15/0,000208**	35.96/0.000000**
SOMS-2 Symptom index	9.72 (7.76)	11.32 (8.95)	5.75 (4.70)	7.68/0.007*	9.88/0.003*
CAR					
-AUCG	50.08 (21.05)	50.73 (22.25)	47.94 (18.18)	0.23/0.631	0.26/0.612
-AUCI	13.45 (14.77)	11.69 (9.57)	14.98 (14.17)	0.22/0.640	0.84/0.365

AUCg: area under the curve with respect to ground; AUCi: area under the curve with respect to increase; BDI-II: Beck Depression Inventory; CAR: cortisol awakening response; SOMS-2: Screening for Somatoform Disorders; TICS: Trier Inventory of Chronic Stress; Notes: *significance at *p* < 0.005; **corrected empirical significance at *p* < 0.00089; method: analysis of variance (ANOVA).

All subjects sampled saliva using Salivettes (Sarstedt, Nümbrecht) on two consecutive days at the time of awakening and 30, 45 and 60 minutes thereafter. The collected saliva samples were analyzed in the biochemical laboratory of the University of Trier using an immunoassay with fluorescence detection [31].

### Statistical Analyses

For all statistical analyses, SPSS 19.0 (Statistical Package for the Social Sciences, SPSS Inc, Chicago, IL) was used. To include the CAR in our analysis, areas under the curve with respect to ground (AUCG) and with respect to increase (AUCI) [32] were calculated for each subject using the single means of the four measurements of the first and the second day. All subjects with negative results of the AUCI were set to zero in order to avoid negative areas and to state that no increase was seen in these subjects. The data derived from the questionnaires as well as the AUCG and the AUCI of the CAR were compared in an one-way ANOVA (analysis of variance) model with the between-subjects factor ‘group’ (‘hyperhidrosis’ and ‘healthy controls’) to detect possible differences between the two groups. Moreover, a dummy variable was created to differ between axillary hyperhidrotics and other hyperhidrotics. Again, three one-way ANOVAs were used to compare axillary hyperhidrotics to other hyperhidrotics, other hyperhidrotics to controls and axillary hyperhidrotics to controls. A χ^2^ model was used to match the sample. The data derived from the HHIQ were evaluated using descriptive statistics. The significance level for all tests was set to *p* = 0.05 and Bonferroni correction was calculated to correct for multiple comparisons. Thus, the corrected significance level for all tests is set to *p* = 0.00089. Furthermore, power analysis was performed using G*Power, version 3.1.0 (http://www.uni-duesseldorf.de/home/Fakultaeten/math_nat/WE/Psychologie/abteilungen/aap/gpower3/download-and-register).

## Results

### Hyperhidrosis Impact Questionnaire

Most of the subjects (47,5%) reported the onset of their hyperhidrosis at an age between 12 and 17 years, 35% were over 17 years old, 10% were between 6 and 11 and 7,5% were under the age of 6 when their excessive sweating began. The body regions most frequently affected were the armpits (50%), followed by the palms (25%), the face (17,5%), the plants (5%) and the back (2,5%).

An overview about the following questionnaire and cortisol data and their statistical analysis is presented in Table 2.

### Trier Inventory of Chronic Stress

The hyperhidrotics of our sample showed significantly higher values for “lack of social recognition” (*p* = 0.001). Uncorrected, four other scales, “social overload” (*p* = 0.019), “excessive demands from work” (*p* = 0.031), “chronic worrying” (*p* = 0.023) and the “chronic stress screening scale” (*p* = 0.010), differed between both groups, reflecting a trend towards more chronic stress in our hyperhidrotic subjects. All other scales did not differ significantly between both groups before or after correction. Comparing only axillary hyperhidrotics to the control group, the hyperhidrotics had significantly higher scales for “excessive demands from work” (*p*  = 0.00025), “lack of social recognition” (*p* = 0.000015), “chronic worrying” (*p* = 0.000156) and the “chronic stress screening scale” (*p* = 0.000031) after Bonferroni correction indicating a higher stress level especially in subjects with axillary hyperhidrosis.

Comparing axillary hyperhidrotics to other hyperhidrotics, and other hyperhidrotics to the control group, no significant differences were found after Bonferroni correction (Table S1). Without correction, axillary hyperhidrotics showed significant higher scales for “social overload” (*p* = 0.048), “work discontent” (*p* = 0.039), “excessive demands from work” (*p* = 0.011), “lack of social recognition” (*p* = 0.031), “chronic worrying” (*p* = 0.003) and the “chronic stress screening scale” (*p* = 0.008) compared to other hyperhidrotics (Table S1).

### Beck Depression Inventory

As measured by the Beck Depression Inventory, 24 out of 40 hyperhidrotics (60%) reached total sum scores equal or greater nine, which represents the screening’s cut-off for an indication of depression. In comparison, 4 out of 40 healthy control individuals (10%) provided a sum equal or greater nine. Overall, the hyperhidrotics showed a significantly higher BDI sum score than the healthy controls (*p* = 0.000208).

Comparing only axillary hyperhidrotics to the control group, an even bigger difference between those groups occurred (*p* = 0.000000) after Bonferroni correction. By comparing axillary hyperhidrotics to other hyperhidrotics, and other hyperhidrotics to the control group, no significant differences were found after correction. Without correction, axillary hyperhidrotics would have a higher BDI-II sum score than other hyperhidrotics (*p* = 0.001) reflecting a trend towards more depressive symptoms in axillary hyperhidrosis compared to other hyperhidrotics (Table S1).

### Screening for Somatoform Disorders

Hyperhidrotics did not show significantly more somatic symptoms compared to the control group. However uncorrected, a trend towards increased somatic symptoms (*p* = 0.007), especially blushing and hot flashes, was reported by the hyperhidrotics. Comparing axillary hyperhidrotics to the controls, axillary to other hyperhidrotics or other hyperhidrotics to the control group, no significant differences were found.

### Cortisol

As shown in Table 2 (and Table S1), the total hormonal output measured by the area under the curve with respect to ground (AUCG) as well as the sensitivity of the HPA measured by the area under the curve with respect to increase (AUCI) showed no significant differences between all groups tested (*p*_all_ ≥ 0.36).

## Discussion

The aim of the current study was to investigate the connection between hyperhidrosis and psychosocial parameters as well as cortisol as a physiological correlate of stress. In line with others [7], [26], the majority of the excessively sweating subjects of our sample reported the onset of their hyperhidrosis in childhood or puberty. These important stages of life are very sensitive for changes and disturbances of the developmental processes of self-esteem and identity [33]. We found that lack of social recognition as one indicator of chronic stress as well as the amount of depressive symptoms is significantly higher in the hyperhidrotics compared to matched healthy controls. Interestingly, axillary sweating had the highest impact on increased TICS and BDI-II scores in the entire hyperhidrotic group, which may result in the higher impairment of axillary hyperhidrotics reported by Hamm and colleagues (2006) [26]. Furthermore, hyperhidrosis causes considerable disruptions of social and professional life, leading to severe limitations of the person’s quality of life [1]. Especially in social situations, excessive sweating, and most notably axillary sweating, might attract attention leading to a stressful condition for the hyperhidrotics. Additionally, emotional processes stimulate or maintain the secretion of sweat resulting in a vicious circle of sweating and social stress described by Sonntag and Ruzicka (2004) [25]. Many people consider sweat as an indication for lacking hygiene and refuse persons who sweat excessively, resulting in the lack of social recognition found in our study. So far, cause and consequence of sweating and stress are still unclear due to their reciprocal interaction.

In our study, we could merely see a trend towards increased somatic symptoms caused by an overactivity of the autonomic nervous system. A dysfunction of the autonomic nervous system is discussed as one reason for somatoform disorders as well as for hyperhidrosis. Thus, Shih and colleagues (1983) postulate an overactive sympathetic nervous system to be causative for hyperhidrosis [11]. Additionally, psychosocial stress might have a maintaining or reinforcing influence on both, excessive sweating and somatoform disorders. Likewise, the disease itself might preserve as a stressor in the wake of chronification [34].

To the best of our knowledge, this is the first study investigating CAR in hyperhidrotics. A multitude of studies, summarized in a meta-analysis by Chida and Steptoe, showed that there is evidence of an increased CAR in individuals reporting chronic stress in general and the lack of social recognition in particular [20]. Although we found an increased lack of social recognition in our hyperhidrotics, we did not detect differences in their cortisol awakening response compared to our control group. However, measurable changes of the CAR are particularly expected in highly to pathologically stressed persons [20]. Indeed, we found that our hyperhidrotic subjects suffer from chronic stress but did not show highly psychopathological symptoms. Thus, our results can be seen congruent with others.

Besides measuring CAR, other methods to approach HPA axis activity could be applied. The Trier Social Stress Test [35] or the socially-evaluated cold-pressor stress test [36] could be used to investigate the cortisol release of hyperhidrotics after acute stress. Another possibility would be a pharmacological intervention, using the dexamethasone suppression test to elucidate the feedback regulation of the HPA axis in excessively sweating people. The communication of cutaneous and systemic HPA axis might be another interesting parameter to look at in further studies on hyperhidrosis. The skin can activate the systemic HPA axis via neural transmission to the brain (hypothalamus), via activation of the pituitary through skin-derived factors, or directly via the activation of the adrenal cortex [8]. Recently, it has been shown that environmental stressors, such as ultra violet radiation, stimulate the production of cutaneous CRH, which can act on the pituitary level of the systemic HPA axis [8]. Likewise, it would be interesting to investigate if the influence of psychosocial stressors is comparable to that of physical environmental stressors. Furthermore, CRH has been investigated as a possible factor in disorders of adnexal skin structures, such as acne vulgaris [8], but not yet in excessive sweating. Thus, this might be a novel aspect in the research of hyperhidrosis.

Beyond the self-reported higher stress levels, the hyperhidrotics of our study manifested more depressive symptoms compared to the control group. The effect size of η^2^ = 0,163 indicates a remarkable effect (conventions after Cohen (1988) [37]). 60% of the hyperhidrotics scored values beyond the threshold for depression in the BDI-II, whereas merely 10% of the controls did. Our results are comparable to a study of Lee and colleagues (2012), who found BDI values similar to ours [17]. In comparison, Ruchinskas and colleagues (2002) detected depressive symptoms in merely 7% of the hyperhidrotics [38] and Weber and co-workers (2005) did not find any difference between hyperhidrotics and the normal population [1]. These different results are justified by various measuring instruments. In our study, the subjects could not be assigned randomly to the two conditions due to the nature of the independent variable. Therefore, a causative conclusion was hardly to draw because hyperhidrosis is not susceptible to manipulation in this quasi-experimental design. However, psychopathological symptoms, for example triggered by self-imposed withdrawal, might rather represent the effect of hyperhidrosis than being its primary cause [38], [39]. Moreover, axillary sweating may constitute an endophenotype genetically and physiologically linked to a higher susceptibility for depression and should be investigated separately. In line with others [40], [41], we found a familial aggregation for different forms of primary hyperhidrosis including axillary and palmar hyperhidrosis (preliminary data). Previously, a heritability of 28% for palmar hyperhidrosis has been shown [42]. Therefore, further investigation of different endophenotypes of hyperhidrosis should include genetic analyses, too.A limitation of our study was the lack of medical diagnosis of hyperhidrosis in most of our subjects, who indicated that they were often too embarrassed by their condition to consult a physician. Therefore, all subjects without prior diagnosis (75%) were tested using a gravimetric examination and the relevant thresholds were exceeded by all of them. Furthermore, we presumed that all excessively sweating subjects had a primary hyperhidrosis. The exclusion of other medical conditions for the explanation of hyperhidrotic symptoms was conducted via personal anamnesis. Questions related to details on the excessive sweating, chronic and acute diseases or disorders and intake of pharmaceuticals were asked to obtain the psychological and physiological status of each subject. Thus, a secondary hyperhidrosis could be excluded with a high probability but not completely.

Additionally, putative expectation effects could occur due to the study design. The hyperhidrotics of the sample participated explicitly because of their disease and the purpose of the study was obvious to them. Hence, they possibly focused on their psychological strain while filling in the questionnaires. Therefore, they might show a higher impact than they would have done under neutral conditions.

Another limitation is the small sample size of our study. However, our sample (N = 80) is comparable to other studies investigating primary hyperhidrosis and would be sufficient to detect a medium effect of f = 0.25 with a significance level α = 0.05 and the power 1- β = 0.60 (conventions after Cohen (1988) [37]). In a bigger sample, some of the other stress scales as well as a higher amount of somatic symptoms could probably be verified. According to the limitations mentioned above, we would like to see our results as an initiation to further investigations in this domain.

In conclusion, we found that individuals with hyperhidrosis suffered from more chronic stress and showed more depressive symptoms compared to the general population but did not show a higher cortisol response. Axillary sweating had a higher impact on stress and depression than all other types of sweating taken together. The knowledge of psychological concomitants of excessive sweating might contribute to a psychotherapeutic treatment in hyperhidrosis and therewith to the quality of life of persons concerned.

## Supporting Information

Table S1 Means and standard deviations (in parentheses) of axillary, other hyperhidrotic and control subjects for different questionnaires and cortisol.
(DOC)
