# Supplementary material for: Elevated Social Stress Levels and Depressive Symptoms in Primary Hyperhidrosis
Source: PLoS One. 2014 Mar 19;9(3):e92412. doi: 10.1371/journal.pone.0092412 (PMC3960246; doi:10.1371/journal.pone.0092412)
Supplement: Table S1 — Means and standard deviations (in parentheses) of axillary, other hyperhidrotic and control subjects for different questionnaires and cortisol. (DOC) [file pone.0092412.s001.doc]

Table S1: Means and standard deviations (in parentheses) of axillary, other hyperhidrotic and control subjects for different questionnaires and cortisol

|  | Control subjects | Axillary hyperhidrotic subjects | Other hyperhidrotic subjects | Difference (Controls – Other hyperhidrotic subjects) F (1, 58) / *p* | Difference (Axillary hyperhidrotic subjects – Other hyperhidrotic subjects) F (1, 38) / *p* |
| --- | --- | --- | --- | --- | --- |
| TICS |  |  |  |  |  |
| -work overload | 50.78 (12.137) | 59.00 (12.689) | 51.86 (9.318) | 0.146 / 0.704 | 4.173 / 0.048* |
| -social overload | 48.30 (7.215) | 53.95 (7.663) | 51.38 (9.410) | 2.115 / 0.151 | 0.883 / 0.353 |
| -pressure to succeed | 47.58 (7.861) | 51.32 (9.086) | 47.76 (7.765) | 0.003 / 0.958 | 1.778 / 0.190 |
| -work discontent | 52.72 (8.111) | 58.79 (7.605) | 53.14 (8.929) | 0.004 / 0.950 | 4.585 / 0.039* |
| -excessive demands from work | 54.33 (9.236) | 64.58 (9.805) | 55.10 (12.365) | 0.075 / 0.784 | 7.120 / 0.011* |
| -lack of social recognition | 48.13 (9.042) | 60.11 (9.189) | 52.33 (12.318) | 2.240 / 0.140 | 5.027 / 0.031* |
| -social tensions | 48.70 (10.957) | 53.21 (13.559) | 50.00 (8.877) | 0.276 / 0.601 | 0.800 / 0.377 |
| -social isolation | 51.75 (10.805) | 56.53 (10.601) | 50.71 (8.168) | 0.221 / 0.640 | 3.814 / 0.058 |
| -chronic worrying | 50.98 (8.300) | 60.89 (9.758) | 51.19 (9.657) | 0.000015 / 0.997 | 9.974 / 0.003* |
| -chronic stress screening scale | 52.28 (7.893) | 63.26 (10.262) | 53.57 (11.487) | 0.247 / 0.621 | 7.851 / 0.008* |
| BDI-II | 4.83 (3.134) | 14.26 (8.925) | 5.95 (5.094) | 0.137 / 0.713 | 13.408 / 0.001* |
| SOMS-2 Symptom index | 5.75 (4.695) | 11.32 (8.951) | 8.10 (6.074) | 2.416 / 0.125 | 1.804 / 0.187 |
| Cortisol Awakening Response |  |  |  |  |  |
| -AUCG | 47.94 (18.18) | 50.73 (22.253) | 49.45 (20.43) | 0.085 / 0.771 | 0.036 / 0.851 |
| -AUCI | 14.98 (14.17) | 11.69 (9.567) | 15.66 (18.20) | 0.057 / 0.812 | 0.724 / 0.400 |

AUCg: area under the curve with respect to ground; AUCi: area under the curve with respect to increase; BDI-II: Beck Depression Inventory; CAR: cortisol awakening response; SOMS-2: Screening for Somatoform Disorders; TICS: Trier Inventory of Chronic Stress; Notes: *significance at *p* < 0.005; **corrected empirical significance at *p* < 0.00089; method: analysis of variance (ANOVA)
